# Analysis of the intramolecular 1,7-lactone of *N*-acetylneuraminic acid using HPLC–MS: relationship between detection and stability

**DOI:** 10.1007/s10719-023-10114-x

**Published:** 2023-04-21

**Authors:** Paolo La Rocca, Ivana Lavota, Marco Piccoli, Federica Cirillo, Andrea Ghiroldi, Giuseppe Ciconte, Carlo Pappone, Pietro Allevi, Paola Rota, Luigi Anastasia

**Affiliations:** 1 Department of Biomedical Sciences for Health, Università degli Studi di Milano, Milan, 20133 Italy; 2 Institute for Molecular and Translational Cardiology (IMTC), San Donato Milanese, Milan, 20097 Italy; 3 Laboratory of Stem Cells for Tissue Engineering, IRCCS Policlinico San Donato, San Donato Milanese, Milan, 20097 Italy; 4 Arrhythmology Department, IRCCS Policlinico San Donato, San Donato Milanese, Milan, 20097 Italy; 5 Faculty of Medicine, University of Vita-Salute San Raffaele, 20132 Milan, Italy; 6 Department of Biomedical, Surgical and Dental Sciences, Università degli Studi di Milano, Milan, 20133 Italy

**Keywords:** Sialic acids, *N*-acetylneuraminic acid, 1,7-lactone, γ-lactone, HPLC–MS analysis

## Abstract

A subclass of the sialic acid family consists of intramolecular lactones that may function as key indicators of physiological and pathological states. However, the existence of these compounds in free form is highly improbable, since they are unlikely to exist in an aqueous solution due to their lability. Current analytical method used to detect them in biological fluids has not recognized their reactivity in solution and is prone to misidentification. However, recent advances in synthetic methods for 1,7-lactones have allowed the preparation of these sialic acid derivatives as authentic reference standards. We report here the development of a new HPLC–MS method for the simultaneous detection of the 1,7-lactone of *N*-acetylneuraminic acid, its γ-lactone derivative, and *N*-acetylneuraminic acid that overcomes the limitations of the previous analytical procedure for their identification.

**Supplementary Information:**

The online version contains supplementary material available at 10.1007/s10719-023-10114-x.

## Introduction

**Fig. 1:**
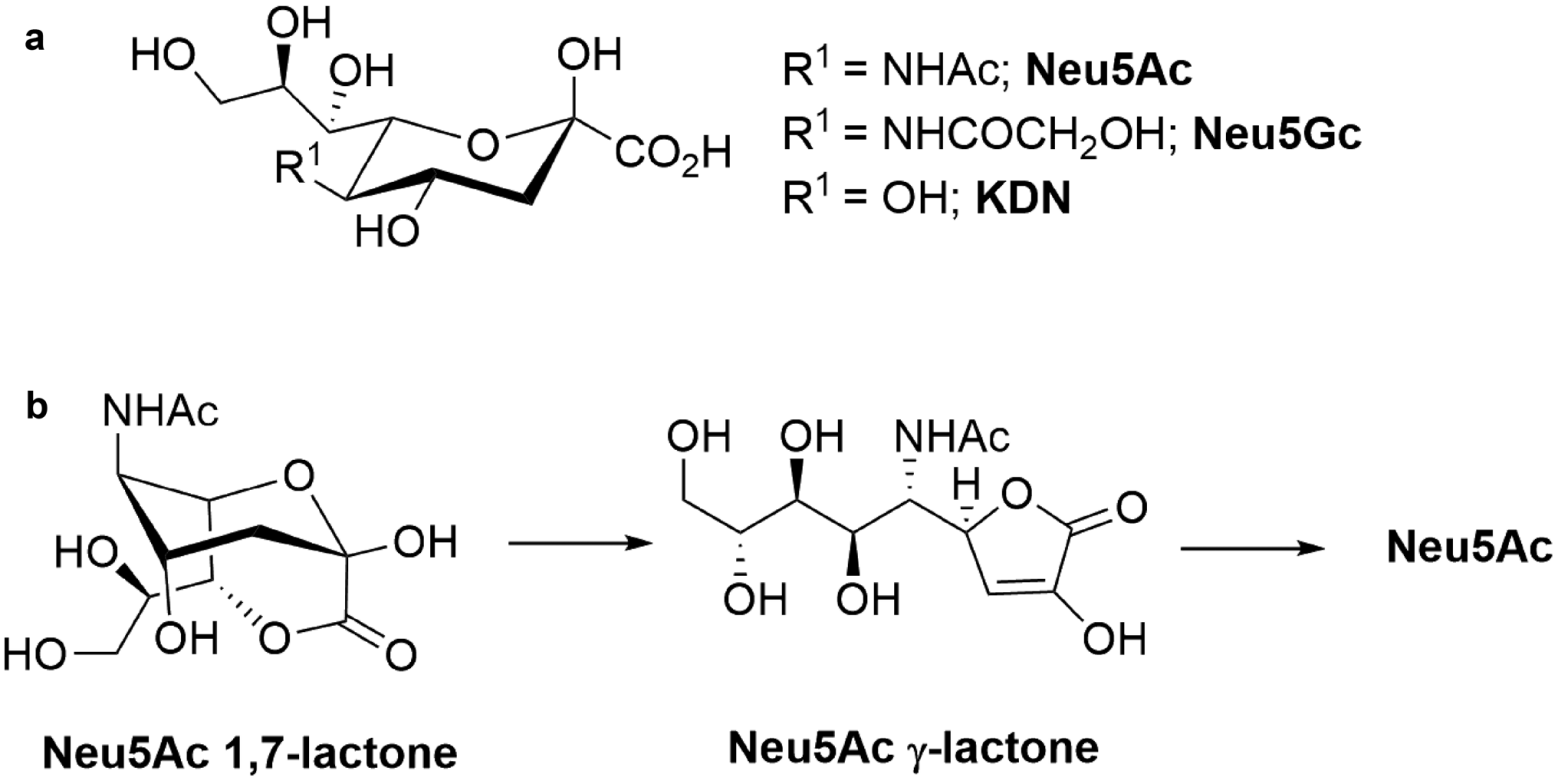
**a** Representation of the three main sialic acids: Neu5Ac, Neu5Gc and KDN; **b** 1,7-lactone of Neu5Ac rearrangement under hydrolytic conditions forming Neu5Ac, through its γ-lactone intermediate [20]

The sialic acids (Sias) are a family of naturally occurring α-keto acids with nine carbon atoms derived from three members, *N*-acetylneuraminic acid (Neu5Ac), *N*-glycolylneuraminic acid (Neu5Gc), and 2-keto-3-deoxy-D-glycero-D-galactonononic acid (KDN, Fig. 1a) [1–7]. The structural diversity of Sias depends on the variety of possible modifications of the hydroxyl groups in these compounds, including the formation of lactams or lactones [8–20]. Sias are typically attached to the non-reducing end of the carbohydrate chain with an α-acetalic bond and are involved in many fundamental physiological and pathological processes [2–6]. They play a critical dual role as a receptor or masking receptor and are involved in the modulation of signal transduction, immunity, growth and differentiation, and recognition processes in pathogenic viral and bacterial infections [21–24]. Thus, these molecules represent the basis of antibacterial and antiviral agents’ synthesis [21–27]. Also, sialylation plays a crucial role in triggering the hypoxic response of cardiac cells [28], which could lead to new therapeutic approaches for ischemic heart disease [29]. There is also evidence that sialylation contributes to the pathology of Brugada syndrome, with sialylation levels being negatively correlated with the severity of the disease [30].

Due to the importance and diversity of Sias, efforts have been made to develop appropriate analytical methods for their identification in biological samples of different origins. The approach that has contributed most to the mapping of these sugars to date is based on the derivatization of their hydroxyl groups as heptafluorobutyrates (HFB) prior to GC–MS analysis [31]. About fifty different sialic acids have been recognized using this method, including some new compounds such as the 1,7-lactone of Neu5Ac (Fig. 1b) [8, 9, 31–43]. However, it was recently revealed that this analytical procedure had some critical flaws [19, 44]. In particular, it was discovered that the 1,7-lactones of Sias that were identified by the method were actually formed by the inadvertent cyclization of the corresponding acids under derivatization conditions. It was also shown that the 1,7-lactones of Sias cannot be determined by this approach because they are decomposed during the first hydrolysis step. Moreover, under acidic and basic hydrolysis conditions used to release Sias from glycoconjugates, the 1,7-lactone of Neu5Ac can rearrange into the more stable γ-lactone and eventually be converted into Neu5Ac (Fig. 1b) [14, 20]. The stability of this 1,7-lactone has been investigated by NMR experiments [12, 14, 44] suggesting that it decomposes slowly in neutral or acidic aqueous solutions at 25 °C as a function of pH, but this evidence has never been directly investigated in biological media.

Therefore, in this work we have developed a new analytical HPLC–MS approach that overcomes the flaws of the GC–MS method and allows the simultaneous detection of underivatized Neu5Ac 1,7-lactone, its γ-lactone, and Neu5Ac using a C_8_ reversed-phase column and [^13^C_3_]Neu5Ac as an internal standard. A stability study was also performed on the different solvents and conditions used for the analysis and purification steps from the biological matrix (plasma). Finally, we have performed experiments in human plasma that completely rule out the possibility that the free 1,7-lactone of Neu5Ac can survive in free form in this biological fluid, even as a more stable γ-lactone.

## Materials and methods

### Materials

All chemicals and solvents used were of analytical grade (purity ≥ 99.9) and purchased from Fisher Scientific (Italy). Deionized water was prepared by filtering water on a Milli-Q Q-POD filtration system from Merck (Darmstadt, Germany). Ultrapure water Optima was purchased from Fisher Scientific (Italy). Solvents and water were sonicated before their use for HPLC analysis. *N*-acetylneuraminic acid (Neu5Ac) was purchased from Carbosynth (Compton, UK). 1,2,3-[^13^C_3_]-*N*-acetylneuraminic acid ([^13^C_3_]Neu5Ac, CAS 1172608–56-8) was purchased from Isotec (Sigma Aldrich). *N*-acetylneuraminic acid 1,7-lactone (Neu5Ac-1,7-lactone) and *N*-acetylneuraminic acid γ-lactone (Neu5Ac-γ-lactone) were prepared in our laboratory following the procedures previously published [12, 16, 20].

### NMR characterization

Nuclear magnetic resonance spectra were recorded at 298 K on a Bruker AM-500 spectrometer equipped with a 5-mm inverse-geometry broadband probe and operating at 500.13 MHz for ^1^H and 125.76 MHz for ^13^C. Chemical shifts are reported in parts per million and are referenced for ^1^H spectra to a solvent residue proton signal (*δ* = 2.50 ppm for DMSO-*d*_6_). The chemical shifts for the spectra collected in D_2_O are referenced to the internal CH_3_OH residue proton signal (*δ* = 3.31 ppm for ^1^H spectra). The ^1^H NMR data are tabulated in the following order: number of protons, multiplicity (s = singlet, d = doublet, t = triplet, br = broad, m = multiplet, app = apparent), coupling constant(s) are given in Hz, and assignment of proton(s).

#### 1,7 lactone of Neu5Ac

^1^H NMR (500.13 MHz, DMSO-*d*_*6*_) 8.21 (1H, d, *J*_NH,5_ = 8.4 Hz, NH), 7.27 (1H, s, OH), 5.43 (1H, br d, *J* = 2.6 Hz, OH), 5.18 (1H, d, *J* = 6.2 Hz, OH at C-8), 4.62 (1H, t, *J* = 5.6, OH), 4.31 (1H, s, H-6), 4.20 (1H, d, *J*_7,8_ = 8.5 Hz, H-7), 3.81 (1H, br s, H-4), 3.74 (1H, d, *J*_5,NH_ = 7.5 Hz, H-5), 3.58 (1H, m, H-9a), 3.53 (1H, m, H-8), 3.45 (1H, dd, *J*_9b,9a_ = 11.0 Hz, *J*_9b,8_ = 5.6 Hz, H-9b), 1.95 (1H, br dd, *J*_3a,3b_ = 14.0 Hz, *J*_3a,4_ = 3.1 Hz, H-3a), 1.88 (3H, s, CH_3_CONH), 1.81 ppm (1H, br d, *J*_3b,3a_ = 14.0 Hz, H-3b). HRMS (ESI negative, *m/z*): calcd for C_11_H_16_NO_8_ [M − H]^–^ 290.0881, found 290.0882. Other physico-chemical properties were identical to those previously reported [12].

#### γ-lactone of Neu5Ac

^1^H NMR (500.13 MHz, D_2_O) 6.20 (1H, d, *J*_3,4_ = 1.9 Hz, H-3), 5.49 (1H, br s, H-4), 4.28 (1H, dd, *J*_5,4_ = 1.9, *J*_5,6_ = 10.4 Hz, H-5), 3.99 (1H, br d, *J*_6,5_ = 10.4 Hz, H-6), 3.79 (1H, dd, *J*_9a,8_ = 2.7, *J*_9a,9b_ = 11.8 Hz, H-9a), 3.72 (1H, ddd, *J*_8,9a_ = 2.7, *J*_8,9b_ = 6.3, *J*_8,7_ = 9.0 Hz, H-8), 3.57 (1H, dd, *J*_9b,8_ = 6.3, *J*_9b,9a_ = 11.8 Hz, H-9b), 3.44 (1H, br d, *J*_7,8_ = 9.0 Hz, H-7), 1.88 ppm (3H, s, NHCOCH_3_). HRMS (ESI negative, *m/z*): calcd for C_11_H_16_NO_8_ [M − H]^–^ 290.0881, found 290.0882. Other physico-chemical properties were identical to those previously reported [20].

### Liquid chromatography

The chromatographic system consists on a Vanquish UHPLC system with a 25 µl sample loop (Thermo Fisher Scientific, San Jose, CA, USA) coupled online to a Q-Exactive Plus mass spectrometer (Thermo Fisher Scientific, Bremen, Germany). LC elution was performed using a Hypersil GOLD aQ 3 μM, 150 mm × 3 mm HPLC C_8_ column (Thermo Fisher Scientific, San Jose, CA, USA). Chromatography was carried out at 25 °C using as mobile phase A water + 0.1% formic acid and as mobile phase B acetonitrile + 0.1% formic at a constant flow rate of 0.250 ml/min (see Table 1 for the method description).

**Table 1:** HPLC method settings

Time (min)	%A	%B
0	100	0
3.5	100	0
5.5	80	20
7.5	80	20
8.5	100	0
12	100	0

### Mass spectrometry

Mass spectrometry analysis was performed using a Q-Exactive Plus Orbitrap mass spectrometer equipped with an Ion Max API source fitted with an electrospray probe (HESI-II) (Thermo Fisher Scientific, Bremen, Germany). The mass spectrometer was set with ESI spray voltage of 2.50 kV, capillary temperature of 320 °C, sheath and auxiliary gas flow of 35 and 10 units, respectively and S-lens RF value of 50%. All spectra were acquired in negative ion mode with a full-scan MS spectra visualizing, from 70 to 400 m*/z* at 70,000 resolution after accumulating to a target value (AGC target) of 1.00e^6^, with maximum accumulation (Max IT) of 100 ms. Successively, a parallel reaction monitoring analysis (PRM) was performed in negative ion mode at a resolution of 17,500, AGC target set at 2.00e^4^ and a maximum IT of 100 ms. The isolation window was set to 1.5 m*/z* and the normalized collision energy (NCE) to a value of 25 and 60. The inclusion list used in PRM analysis consists of *N*-acetylneuraminic acid (308.09870 *m*/*z*), [^13^C_3_]*N*-acetylneuraminic acid (311.11605 *m*/*z*), *N*-acetylneuraminic acid 1,7-lactone and *N*-acetylneuraminic acid γ-lactone (290.08814 *m*/*z*). Calibration was performed before the analysis using the Pierce™ Negative Ion Calibration Solutions (Thermo Fisher Scientific Bremen, Germany).

Data acquisition was carried out by the Excalibur software (version 4.4.16.14, Thermo Fisher Scientific) and raw files elaboration was accomplished using Freestyle 1.8 SP2 software (Thermo Fisher Scientific).

### Standard stock and working solutions

Standard stock solutions of Neu5Ac, [^13^C_3_]Neu5Ac, Neu5Ac-1,7-lactone and Neu5Ac-γ-lactone were prepared accurately dissolving 1 mg of the standard compound in 1 ml of deionized ultrapure water (LC–MS grade) for Neu5Ac and [^13^C_3_]Neu5Ac, and acetonitrile (LC–MS grade) for the two lactones, obtaining solutions with a concentration of 1000 mg/L. The stock solutions were aliquoted and stored at -20 °C. Neu5Ac and [^13^C_3_]Neu5Ac were found unchanged after up to 1 month, unlike the 1,7-lactone stock solution that showed, after 2 weeks, a 35% of degradation. 10 mg/L working solution of Neu5Ac and [^13^C_3_]Neu5Ac were prepared making a 1:100 dilution of the stock solution in ultrapure water; 50 mg/L Neu5Ac-1,7-lactone and Neu5Ac-γ-lactone solutions were prepared making 1:20 dilution of their stock solutions in acetonitrile (LC–MS grade).

### Stability test

90 µL of a selected solvent: CH_3_CN or pure water, or CH_3_CN/water (1:1, v/v) is spiked with 5 µL of Neu5Ac 1,7-lactone stock solution and 5 µL of [^13^C_3_]Neu5Ac 10 mg/L at 0 °C and vortexed for 30 s. Successively, a series of injections for HPLC–MS analyses were performed at different times (0, 1 h, 3 h, 5 h and after 24 h or 72 h). Notably, the stability has been evaluated both at RT and 4 °C. In addition, to test the stability to the drying process, a replicate of the sample at t = 0 has been incubated in the SpeedVac at 40 °C for 40 min, then resuspended in CH_3_CN/water (1:1, v/v) mixture and injected for the analysis.

### MS method sensitivity

Some sequential dilutions of Neu5Ac 1,7-lactone have been prepared in CH_3_CN starting from the 50 mg/L working solution and stored at -20 °C until use. Successively, 5 µL of the analyte and 5 µL of 10 mg/L [^1^^3^C_3_]Neu5Ac standard have been added to 90 µL of a CH_3_CN/water, 1:1 v/v mixture (to ensure the complete solubilization of both the molecules) to obtain the final 1,7-lactone concentrations of 5, 1, 0.5, 0.25, 0.1 mg/L. Acquisitions using full scan and PRM modes have been collected. All the steps have been performed at 4 °C in accordance with the results reported and discussed in the “Results and discussion” session.

### Sample preparation

The sample preparation was performed using three different matrices/media: ultrapure water, surrogate matrix (BSA) or plasma. In particular, for the last two, was used a 5% BSA solution prepared in ultrapure water and a pooled blank human plasma stored at -80 °C and melted to room temperature. All the preparation steps were executed operating rigorously in ice. Regardless of the matrix, 5 µL of [^13^C_3_]Neu5Ac 10 mg/L and 5uL of 1,7-lactone 50 mg/L (or 5uL of water for the controls) were added to 100uL of the related matrix and the final solution was vortexed for 1 min. Then, 100 μL of deionized ultrapure water was added, followed by a 30 s vortex (this step was exclusively applied to BSA and plasma matrices). Successively, after the addition of 300 μL of acetonitrile (to precipitate proteins), all samples were vortexed for another 1 min and, finally, centrifugated at 15,000 g × 5 min. The water sample was directly injected for HPLC–MS analysis (path B, Fig. 5) or dried at 40 °C using the Savant SpeedVac SPD120 (Thermo Fisher Scientific) and then dissolved in CH_3_CN/water (1:1, v/v) before analysis (path A, Fig. 5). On the other hand, BSA and plasma samples were subjected to an additional filtration step of the supernatant using a 0.22 µM syringe filter (Euroclone, Milan, Italy). In both cases, as just explained for the water sample, the filtrate was directly injected for analysis (path B, Fig. 5) or dried at 40 °C using the Savant SpeedVac SPD120 (Thermo Fisher Scientific), and dissolved in CH_3_CN/water (1:1, v/v) before analysis (path A, Fig. 5).

### Statistical analysis

GraphPad Prism 8 (GraphPad Software Inc., La Jolla, CA) was used for graphical display and statistical analysis of the obtained data. The results were presented as the mean of two or more independent experiments carried out in duplicate. An unpaired *t*-test has been performed. Values of p < 0.05 were considered statistically significant.

## Results and discussion

### Optimization of HPLC–MS conditions

Initially, we established the best chromatographic and MS conditions for efficient detection and monitoring of the 1,7-lactone of Neu5Ac and its rearranged derivatives. To correctly identify the 1,7-lactone of Neu5Ac in biological matrices, a mild HPLC–MS method was developed to overcome the the instability of the compound in various polar solvents and its tendency to convert to the parent γ-lactone and eventually to the free Neu5Ac [20]. In this regard, the method had to discriminate between the 1,7-lactone of Neu5Ac, its γ-lactone, and Neu5Ac. Different chromatographic conditions could be tested and optimized thank to the availability of the pure authentic standard (> 95% NMR purity) for both lactones (obtained according to our synthetic procedures [12, 16, 20], see ^1^H NMR tabulation in the “Materials and Methods” section), together with the commercial Neu5Ac and its isotopologue ([^13^C_3_]Neu5Ac) used as an internal standard. The Thermo Fisher C8 aQ column proved to be the best choice, allowing the separation of the three analytes with high resolution and very short elution time (see Table 1). While the HPLC elution was performed using only water, the presence of variable amounts of CH_3_CN (or other solvents) used to resuspend the sample affects the retention time of the analytes, as observed for the two lactones (see Table 2). On the contrary, Neu5Ac and [^13^C_3_]Neu5Ac maintained a constant retention time of 3.27 ± 0.01 min with all solvents tested.

**Table 2:** Retention time (mean ± st.dev.) of 1,7-lactone and γ-lactone standards resuspended in different solvent mixtures

Solvent mix	1,7 lactone RT (min)	γ-lactone RT (min)
CH_3_CN	4.10 ± 0.01	4.27 ± 0.02
CH_3_CN:H_2_O (3:1)	4.12 ± 0.01	4.35 ± 0.02
CH_3_CN:H_2_O (3:2)	4.13 ± 0.01	4.40 ± 0.02
CH_3_CN:H_2_O (1:1)	4.15 ± 0.01	4.45 ± 0.02
H_2_O	4.18 ± 0.01	4.64 ± 0.02

**Fig. 2:**
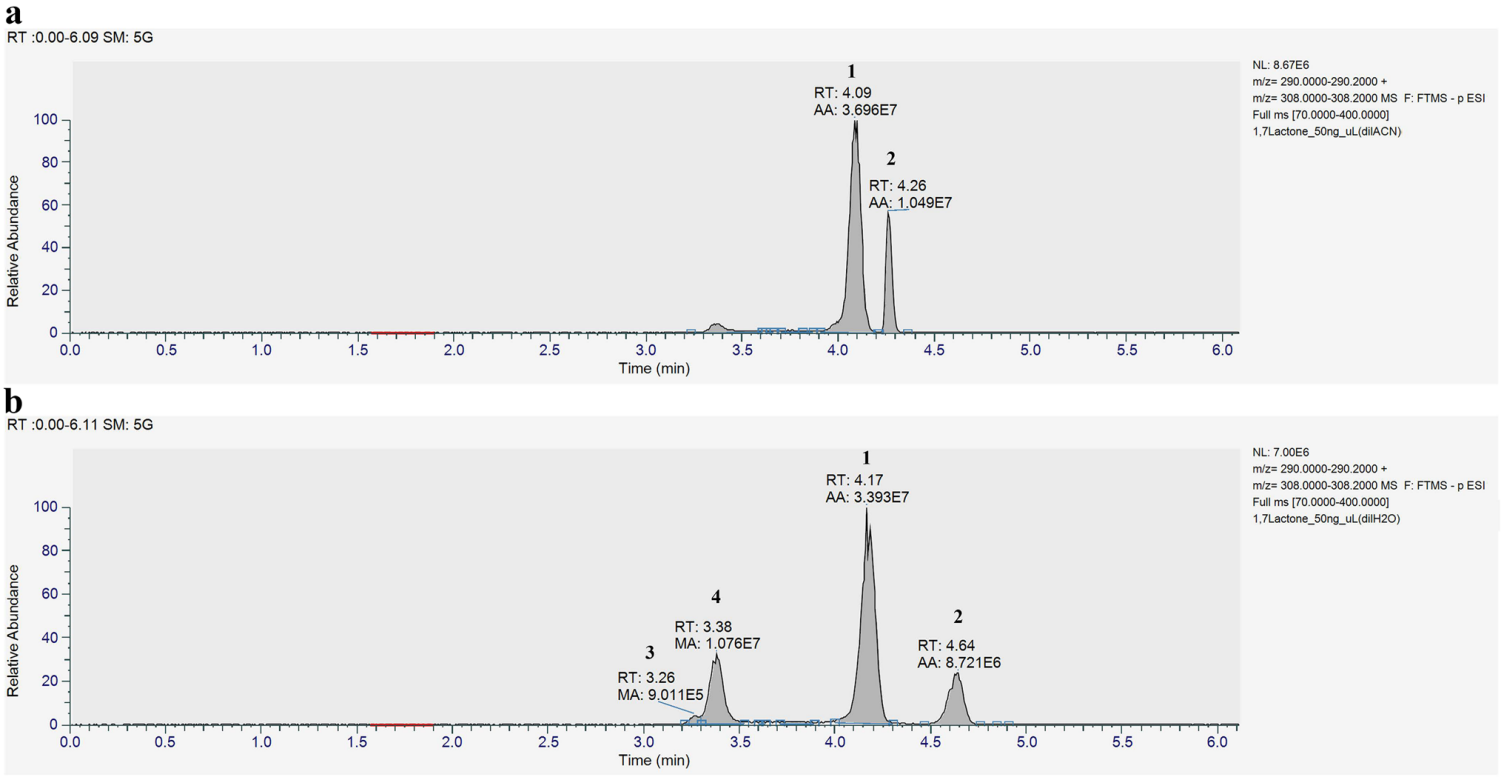
Full Scan analyses of a 50 mg/L Neu5Ac 1,7 lactone solution in ultrapure CH_3_CN (**a**) or ultrapure water (**b**). The mass range of 290.0000–290.2000 and 308.0000–308.2000 have been extracted. Peak **1** (Neu5Ac 1,7-lactone); peak **2** (Neu5Ac γ-lactone); peak **3** (Neu5Ac) and peak **4** (unknown intermediate)

Upon injection of a pure 50 mg/L Neu5Ac 1,7-lactone solution (> 95%, NMR purity) obtained by a 1:20 dilution of the 1 mg/ml stock solution in pure CH_3_CN, two separate peaks of the same molecular weight were detected in the negative electrospray full scan analysis (Fig. 2a). The major peak (**1**) at 4.09 min corresponded to the intact 1,7-lactone, while the minor peak (**2**) at 4.27 min corresponded to the γ-lactone, which conceivably formed from the 1,7-lactone due to the presence of water. This hypothesis was confirmed after injection of pure standard γ-lactone (50 mg/L) solubilized in CH_3_CN, yielding a single peak that eluted at 4.27 (Supplementary Fig. S1a).

A similar procedure was used by injecting a pure 50 mg/L Neu5Ac 1,7-lactone solution diluted in water (Fig. 2b) instead of CH_3_CN. In this case, two additional peaks (**3** and **4**) were observed, distinct from those of 1,7-lactone (**1**) and γ-lactone (**2**) (Fig. S1b). Peak **3** corresponded to Neu5Ac (Fig. S1c), whereas peak **4** could correspond to an unstable intermediate formed in the presence of water, possibly representing a transition structure between the γ-lactone and Neu5Ac.

**Fig. 3:**
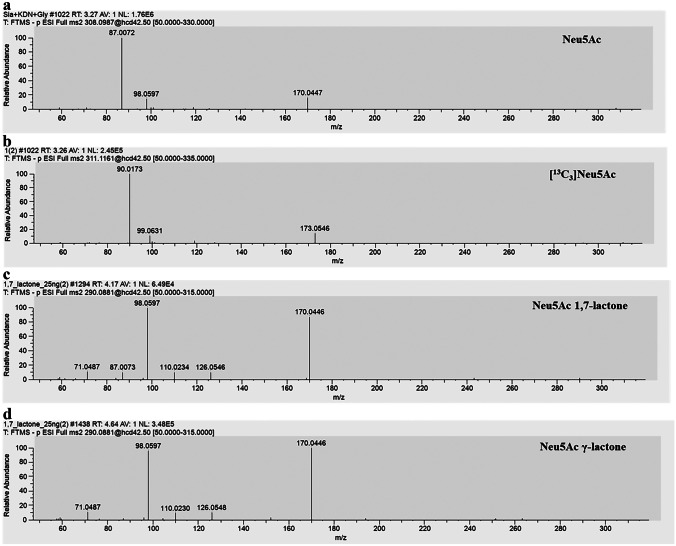
Fragmentation pattern obtained in the PRM analyses of the different precursor ions: Neu5Ac (**a**), [^13^C_3_]Neu5Ac (**b**), 1,7 lactone (**c**) and γ-lactone (**d**)

The method also provided parallel reaction monitoring (PRM) analysis in negative ion mode with an inclusion list consisting of 308.09870 *m*/*z* (Neu5Ac), 311.11605 *m/z* ([^13^C_3_]Neu5Ac), and 290.08814 *m*/*z* (Neu5Ac 1,7-lactone and Neu5Ac γ-lactone). This type of acquisition is based on the isolation of the different precursor ions and their successive fragmentation with a specific collision energy (NCE = 42.5), resulting in a specific fingerprint (Figs. 3 and S2). Under these conditions, the 308.1 *m*/*z* peak corresponding to Neu5Ac shows a fragmentation pattern consisting of three main fragments: 87.0, 170.0 *m*/*z*, and 98.1 *m*/*z*, as reported in the literature [45]. In most cases, the signal of the intact precursor ion (308.1 *m*/*z*) was found.

Moreover, the two peaks corresponding to the molecular weight of two lactones (290.1 *m*/*z*) showed a highly overlapping fragmentation pattern (Fig. 3c and d): 98.1, 170.0 *m*/*z* as the most abundant fragments and 71.0, 110.0, and 126.0 *m*/*z* as the less abundant ones. However, the fragment with 87.0 *m*/*z* appeared to be typical of the 1,7-lactone allowing to discriminate it from the γ-lactone, although the same fragment is also typical of sialic acid. Also in this case, the signal of the intact precursor ion (290.1 *m*/*z*) was occasionally detected.

The sensitivity of the MS method was evaluated (see Materials and Methods for full details). Briefly, lower amounts of 1,7-lactone (5, 1, 0.5, 0.25, 0.1 mg/L) were injected in the presence of 10 mg/L [^1^^3^C_3_]Neu5Ac as an internal standard dissolved in CH_3_CN/water (1:1 v/v mixture, in agreement with the stability data reported in the next paragraph) and analyzed using the method settings reported previously. The resulting curve (y = a + bx; b = 0.400888; a = -0.01105), calculated by linear regression using the peak areas in full-scan mode and weighted by a factor of 1/x^2^, gave an R^2^ value of 0.990 (Table S1-S3). The concentration of 0.10 mg/L resulted the lowest detectable with full-scan mode acquisition. Otherwise, a general loss of sensitivity in the PRM mode was observed and the lowest detected 1,7-lactone concentration corresponded to 0.25 mg/L.

### Stability of the 1,7-lactone of Neu5Ac

**Fig. 4:**
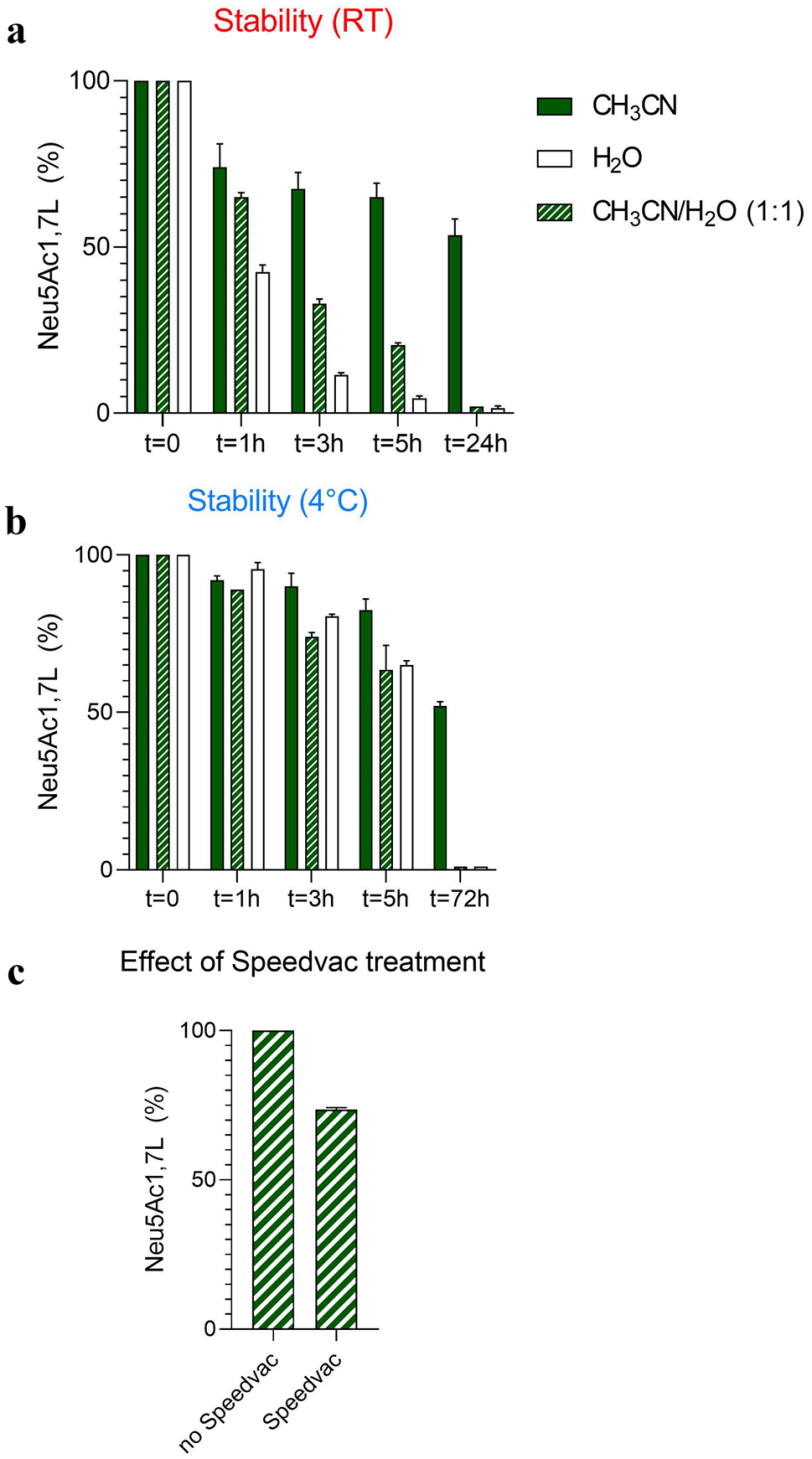
Stability of 1,7 lactone in different solvents: pure CH_3_CN, pure water, and CH_3_CN/water, 1:1 v/v mixture, during time, at RT (**a**) and at 4 °C (**b**). Stability of 1,7 lactone dissolved in CH_3_CN/water, 1:1 v/v mixture after SpeedVac treatment, 40 °C × 40 min (**c**). 1,7 lactone quantification has been expressed as percentage of 1,7-lactone peak areas normalized for the [^13^C_3_]Neu5Ac and assigning the 100% to the 1,7-lactone levels at t = 0. Each value represents the mean of two independent experiments carried out in duplicate

Evaluation of the stability of 1,7-lactone was necessary to select the appropriate solvent for sample preparation and to understand its behavior in biological matrices. We performed this study using three different solvent systems (pure CH_3_CN, pure water, and a CH_3_CN/water mixture, 1:1 v/v) at two different temperature conditions: RT and 4 °C. Specifically, a 50 mg/L solution of Neu5Ac 1,7-lactone was used, corresponding to a 1:20 dilution of the 1 mg/L stock solution under these different conditions, and the recordings were performed at different times (Fig. 4a and b).

As expected, the Neu5Ac 1,7-lactone showed the highest stability in CH_3_CN. Indeed, a 35% decrease in the amount of this lactone was observed after 5 h at RT. On the other hand, a drastic reduction (88%) occurred after only 3 h in water, leading to the simultaneous formation of a high percentage of γ-lactone, Neu5Ac, and unknown intermediates. Remarkably, complete degradation of the 1,7-lactone level occurred after 5 h in water (95%).

Otherwise, in all cases, the standard solutions showed relatively higher stability when stored in the autosampler at 4 °C (Fig. 4b). In particular, only 20% degradation in water and 10% in CH_3_CN was observed after 3 h and 35% and 17% after 5 h, respectively. Interestingly, only 50% degradation of the 1,7-lactone was observed exclusively in CH_3_CN after 72 h at 4 °C and after 24 h at RT. The behavior of the molecule in CH_3_CN/water, 1:1 v/v mixture at 4 °C gave a similar result as in water.

Overall, these data increased our understanding of the stability of this molecule, resulting in the practice of always preparing samples at 4 °C. At this temperature, compound degradation in the presence of water is minimal. Moreover, this observation led us to conclude that it is best to work at 4 °C even during the resuspension of the sample and to dissolve it in a CH_3_CN/water mixture (1:1, v/v), since this allows the simultaneous analysis of both the 1,7-lactone and Neu5Ac, the latter not being very soluble in pure CH_3_CN.

Since an important step in sample preparation is drying the sample with a SpeedVac session at 40 °C, the stability of the 1,7-lactone was also tested under these conditions in the presence of a mixture of CH_3_CN/water (1:1, v/v) to simulate sample preparation conditions where both CH_3_CN and water are present. Interestingly, this procedure appears to have a minimal but non-negligible effect on the stability of Neu5Ac 1,7-lactone, as it was reduced by 30% after treatment (Fig. 4c).

We have presented the stability profile of this compound, confirming its instability observed in previous NMR studies [12, 14, 44]. This process was accelerated in water as compared to CH_3_CN. Otherwise, the degradation was minimal when the process was performed at low temperature (4 °C). The limited degradation during the SpeedVac drying process, confirmed the possibility of using this procedure during the sample preparation protocol without compromising the identification of 1,7-lactones.

### Detection of 1,7 lactone in biological samples

**Fig. 5:**
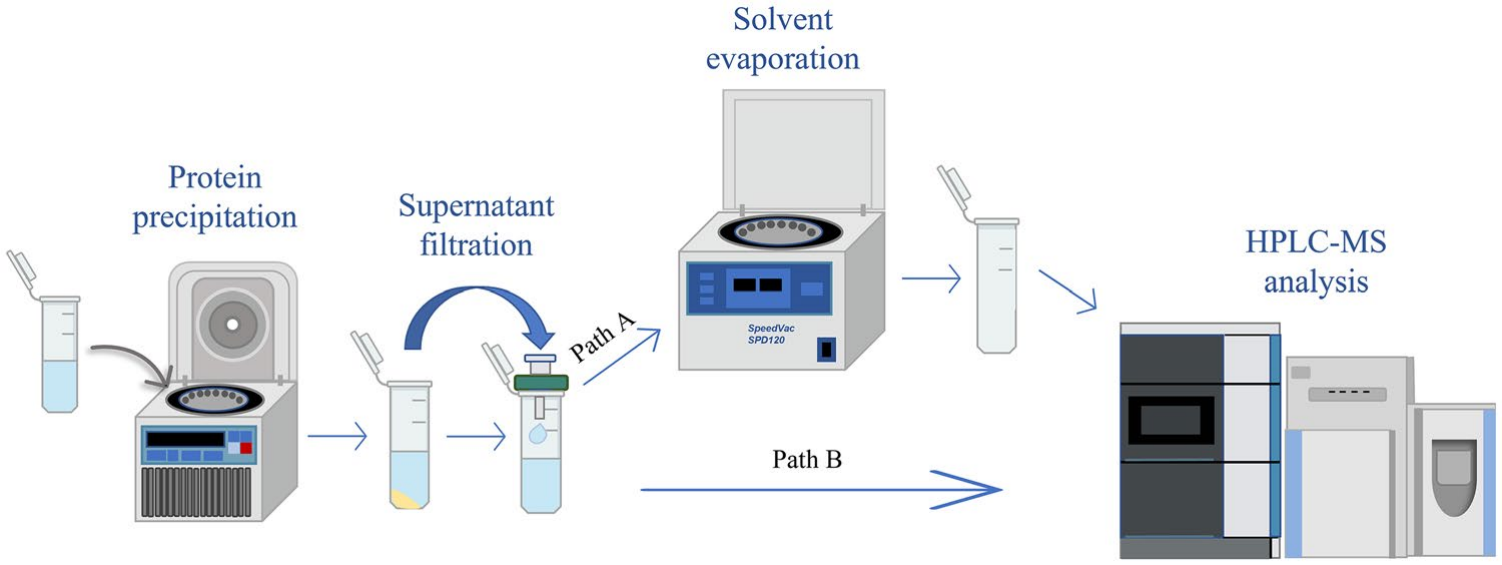
Schematic representation of the two simplified purification methods adopted to preserve 1,7 lactone stability (path A, SpeedVac drying session; path B, direct injection of supernatant)

Finally, biological samples (plasma matrix) were analyzed using this analytical method with the ultimate goal of detecting the 1,7-lactone. A critical step prior to HPLC–MS analysis is sample preparation: the classical purification methods for the isolation of Sias from plasma media are protein precipitation [46], coupled or not with different approaches based on distinctive techniques, such as column purification [31, 47, 48]. Due to the instability of lactones in aqueous solutions, a fast and simplified protocol had to be chosen. For this reason, we selected a simplified purification based solely on protein precipitation, drying of the sample with SpeedVac, and subsequent resuspension in a solvent prior to analysis (Fig. 5, path A). Alternatively, direct injection of the supernatant from precipitation into HPLC was performed (Fig. 5, path B).

#### Purification protocol performance in water

**Fig. 6:**
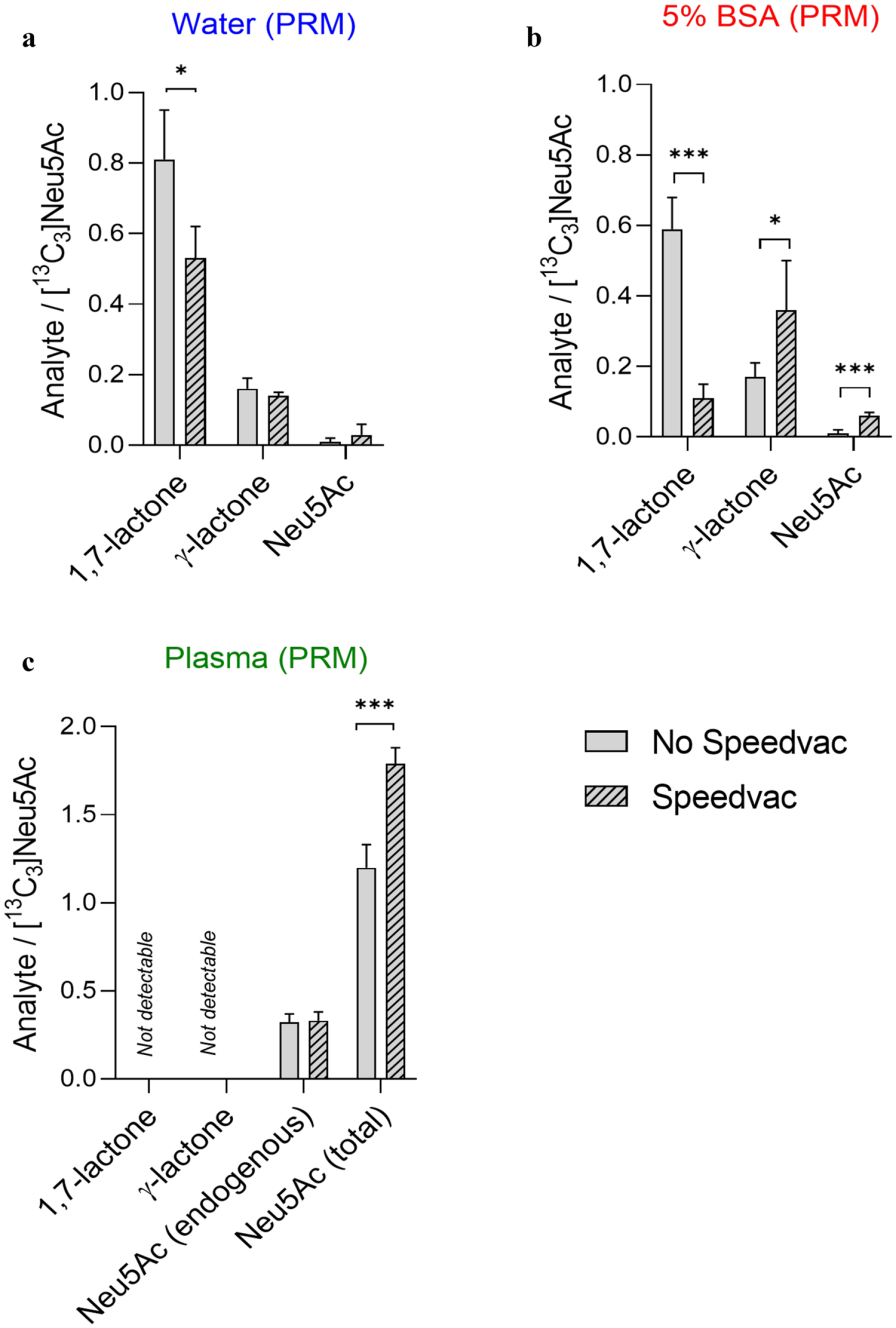
Variations of the 1,7-lactone of Neu5Ac, γ-lactone of Neu5Ac and Neu5Ac levels before and after SpeedVac treatment considering the sample processing in different media/matrices: **a** water, **b** 5% BSA, **c** plasma. The levels of analyte have been expressed as the ratio between the analyte and the [.^13^C_3_]Neu5Ac peak areas from the PRM analysis. For plasma also the endogenous levels of Neu5Ac have been reported. Each value represents the mean of three independent experiments carried out in duplicate (see Table S4). A p-value < 0.05 has been considered statistically significant; *p-value < 0.05; ***p-value < 0.0005

First, the purification protocol was simulated in pure water to investigate the feasibility of the different steps in a simplified aqueous environment, using Neu5Ac 1,7-lactone and [^13^C_3_]Neu5Ac as an internal standard. Specifically, 5 µL of 50 mg/L pure (> 95%) 1,7-lactone was dissolved in ultrapure water, and the purification protocol (see the detailed method in the “Materials and Methods” section) was carefully applied. After protein precipitation simulation, the sample was directly injected for HPLC–MS analysis or dried at 40 °C using Savant SpeedVac SPD120 and then resuspended in CH_3_CN/water (1:1, v/v) before analysis. As shown in Fig. 6a, a large amount of 1,7-lactone was recovered, accompanied by a small amount of γ-lactone with no traces of Neu5Ac in the directly injected samples. Otherwise, only a small but significant decrease of 30–35% in the amount of 1,7-lactone was detected after SpeedVac treatment in both full scan (Fig. S3a) and PRM (Fig. 6a). In addition, only traces of Neu5Ac were found. These results indicate that all purification conditions described in the method are compatible with Neu5Ac 1,7-lactone detection. In particular, this experiment confirmed that performing all steps (except evaporation of the solvent) at 4 °C allows obtaining the 1,7-lactone.

#### Purification protocol in surrogate matrix

The purification method was then applied to a surrogate matrix spiked with Neu5Ac 1,7-lactone (and [^13^C_3_]Neu5Ac as an internal standard). This experiment was key to understanding the possible effects of the protein environment on the stability of our analyte. A 5% BSA solution (pH 7.2/7.3) was chosen as the plasma surrogate matrix, as described in the literature [48]. Specifically, 50 mg/L of pure (> 95%) 1,7-lactone was dissolved in 5% BSA and the purification protocol was carefully performed. A modest decrease in 1,7-lactone concentration (17–27%) was registered (Figs. 6b and S3b), as compared to the previous experiment in water by direct injection in HPLC–MS. On the other hand, a very significant decrease (75–80%) was observed after SpeedVac treatment. Meanwhile, the content of γ-lactone increased (doubled) in a statistically significant manner. These experiments suggest that the 1,7-lactone in the surrogate matrix decreases significantly, and its degradation is accelerated by SpeedVac treatment at 40 °C. In this case, it might be practical to directly detect the γ-lactone, which is present at higher concentrations with an analyte/[^13^C_3_]-Neu5Ac ratio of 0.30 in full scan analysis and 0.36 in PRM. This evidence definitively confirmed the instability of 1,7-lactone, at least in its free form, and suggested that 1,7-lactone-derived γ-lactone should be sought as a potentially more suitable biomarker because it is more stable in the surrogate matrix, especially in a protein-rich environment with a pH equivalent to that of plasma.

#### Purification protocol in plasma

Finally, the purification protocol was applied to five different human plasma samples. Samples were spiked with 50 mg/L 1,7-lactone (and [^13^C_3_]Neu5Ac as a standard), and the purification protocol was applied. Surprisingly, no traces of 1,7- or γ-lactone were found in all experiments performed under either condition (40 °C SpeedVac and direct injection after precipitation) (Figs. 6c and S3c). Remarkably, a significant fourfold increase in Neu5Ac levels was registered as compared with endogenous plasma levels (Fig. 6c), confirming that 1,7-lactone fully converts in Neu5Ac in plasma, which passes through the γ-lactone derivative or into undefined intermediates. Of note, a significant increase was observed after treatment with SpeedVac, whereas endogenous Neu5Ac levels remained constant. These data suggest that some unknown labile intermediates could probably survive without this treatment, but they are immediately and completely converted to Neu5Ac after the drying process.

In light of these data, it seems that it is very difficult to detect this metabolite in this biological fluid, not even as an intermediate γ-lactone; it is likely that it converts rapidly to Neu5Ac through a variety of unstable intermediates. All assumptions about 1,7-lactone's existence and its behavior in the biological matrix are purely speculative. Therefore, it is likely that the complex protein and enzyme environment in plasma greatly affect the stability of both lactones, which means 1,7-lactone, if it exists, can only be detected if glycoconjugates are present as its more stable form.

## Conclusion

In this work, we have developed an HPLC–MS method that allows the simultaneous separation of the 1,7-lactone of *N*-acetylneuraminic acid, its γ-lactone derivative, and *N*-acetylneuraminic acid without derivatization. The method overcomes the limitations of existing analytical procedures that lead to the misidentification of these compounds. In addition, the best conditions to keep the 1,7-lactone stable in solution were investigated. Nevertheless, only Neu5Ac could be detected in plasma, suggesting that the free 1,7-lactone cannot be identified in this biological fluid, even in its more stable γ-lactone intermediate.

## Supplementary Information

Below is the link to the electronic supplementary material.

Supplementary file1 (DOCX 754 KB)

## Data Availability

The authors declare that the data supporting the findings of this study are available within the article and in supplementary information files.
