# Supplementary material for: Analysis of the intramolecular 1,7-lactone of N-acetylneuraminic acid using HPLC–MS: relationship between detection and stability
Source: Glycoconj J. 2023 Apr 21;40(3):343–54. doi: 10.1007/s10719-023-10114-x (PMC10202997; doi:10.1007/s10719-023-10114-x)
Supplement: Supplementary file 1 — Supplementary file1 (DOCX 754 KB) [file 10719_2023_10114_MOESM1_ESM.docx]

Supplementary Material

**Analysis of the intramolecular 1,7-lactone of *N*-acetylneuraminic acid using HPLC-MS: relationship between detection and stability**

Paolo La Rocca, ^#,°^ Ivana Lavota,^°^ Marco Piccoli,^∥,°^ Federica Cirillo,^∥,°^ Andrea Ghiroldi,^∥,°^ Giuseppe Ciconte,^°,&^ Carlo Pappone, ^°,&,‡^ Pietro Allevi, ^†^ Paola Rota,*^, †,°^ Luigi Anastasia*^,‡,∥,°^

^#^ Department of Biomedical Sciences for Health, Università degli Studi di Milano, 20133 Milan, Italy.

^°^ Institute for Molecular and Translational Cardiology (IMTC), 20097 San Donato Milanese, Milan, Italy

^∥^ Laboratory of Stem Cells for Tissue Engineering, IRCCS Policlinico San Donato, 20097 San Donato Milanese, Milan, Italy.

^&^Arrhythmology Department, IRCCS Policlinico San Donato, 20097 San Donato Milanese, Milan, Italy

^‡^ Faculty of Medicine, University of Vita-Salute San Raffaele, 20132 Milan, Italy. E-mail: luigi.anastasia@hsr.it

^†^ Department of Biomedical, Surgical and Dental Sciences, Università degli Studi di Milano, 20133 Milan, Italy. E-mail: paola.rota@unimi.it

| **Contents** | **Page** |
| --- | --- |
| Full Scan analyses of a 50 mg/L Neu5Ac γ-lactone solution in different solvents (Figure S1) | S2 |
| PRM analyses of a 50 mg/L Neu5Ac 1,7-lactone solution in different solvents (Figure S2) | S2 |
| Table S1 | S3 |
| Table S2 | S3 |
| Table S3 | S3 |
| Table S4 | S4 |
| Variations of the 1,7-lactone of Neu5Ac, γ-lactone of Neu5Ac and Neu5Ac levels before and after SpeedVac treatment (Full scan mode; Figure S3) | S4 |


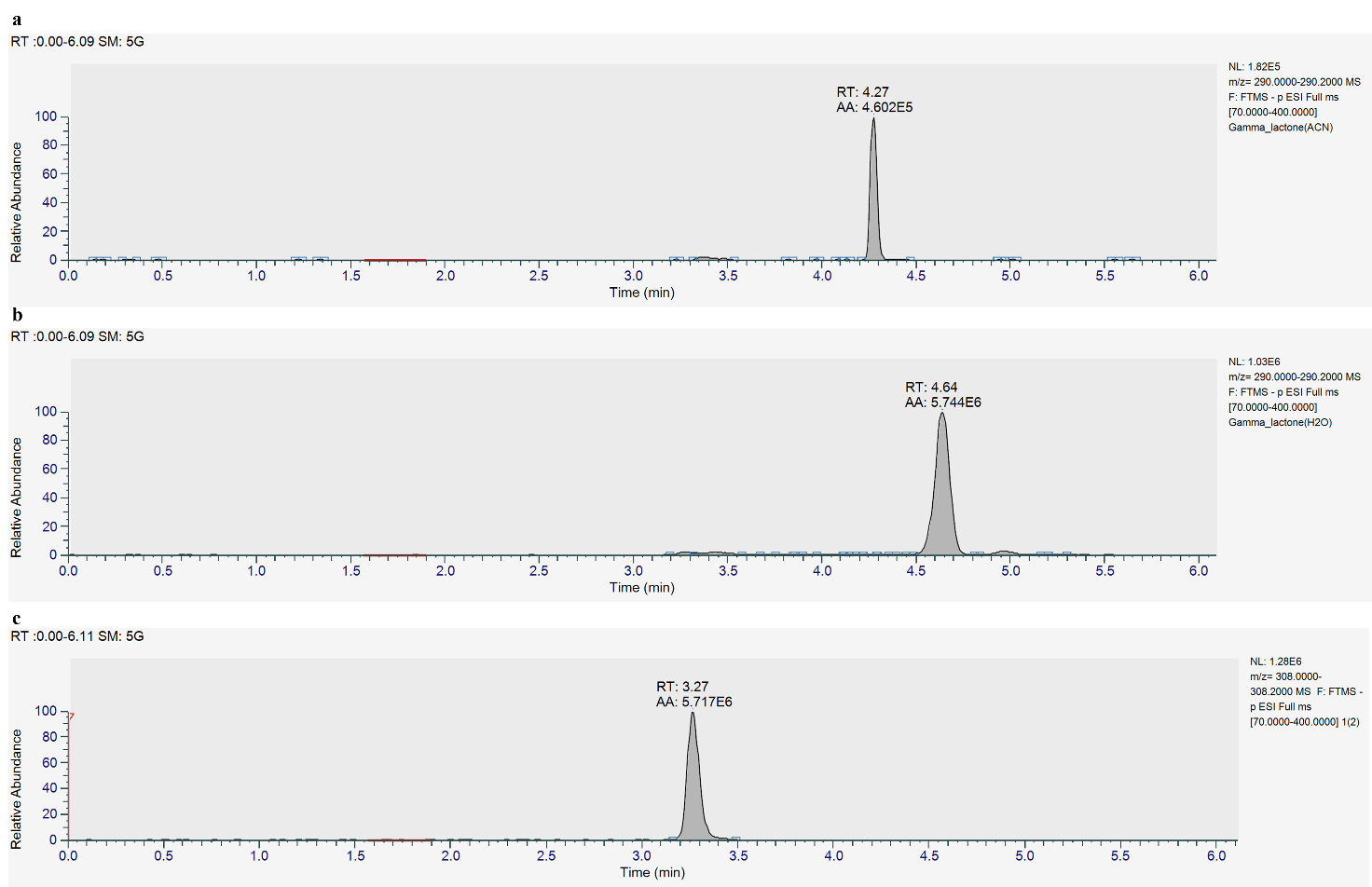


**Fig.S1** Full Scan analyses of a 50 mg/L Neu5Ac γ-lactone solution in ultrapure CH_3_CN (**a**) or ultrapure water (**b**) and Neu5Ac in ultrapure water (**c**). The mass range of 290.0000-290.2000 and 308.0000-308.2000 have been extracted

**1**

**2**


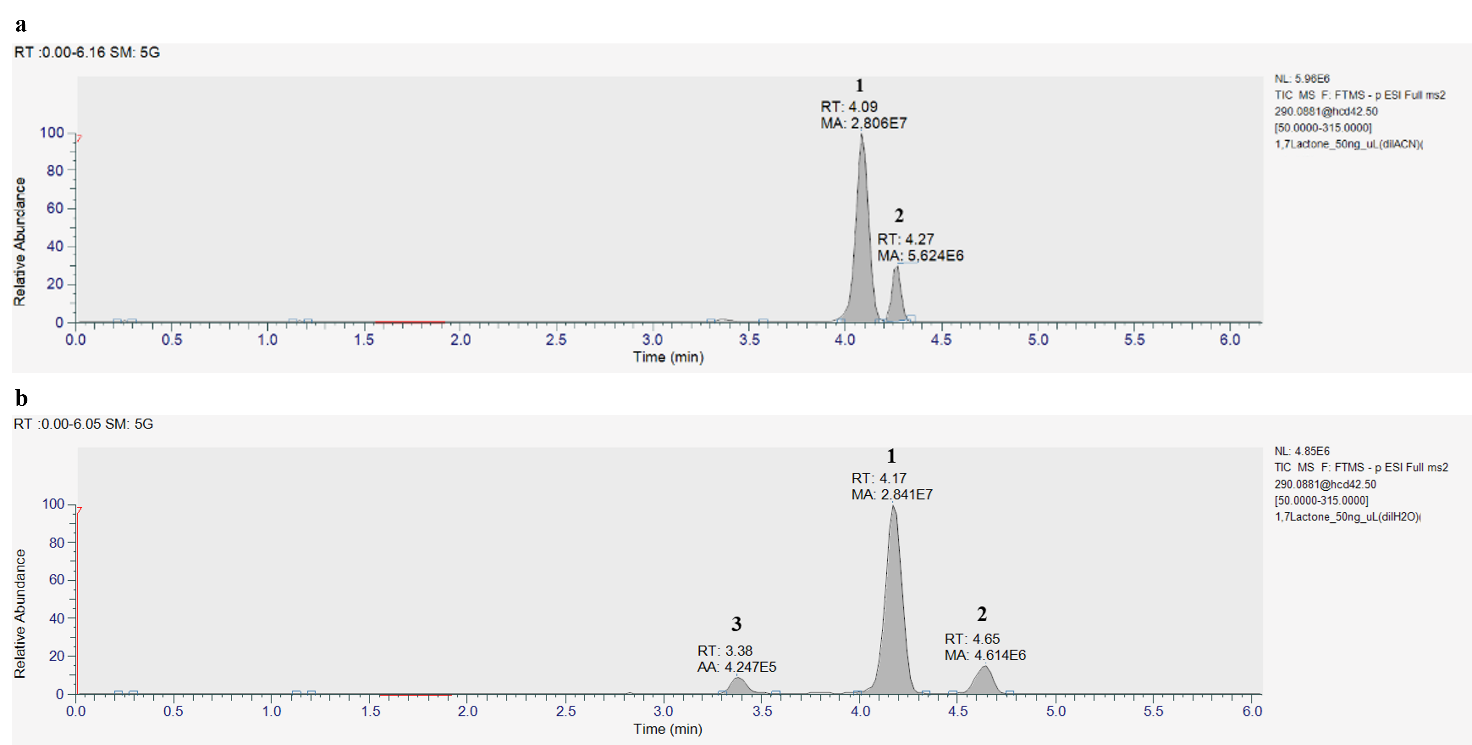


**Fig.S2** PRM analyses of a 50 mg/L Neu5Ac 1,7-lactone solution in ultrapure CH_3_CN (**a**) or ultrapure water (**b**). The precursor ion mass of 290.0881 is herein shown

**Table S1** Peak area values in full scan mode referred to the different 1,7-lactone concentrations (in CH_3_CN/water, 1:1 v/v mixture) injected and normalized for [^13^C_3_]Neu5Ac. Each value represents the mean of two independent experiments carried out in duplicate ± standard deviation.

| **nominal concentration (mg/L)** | **1,7-lactone** | **[^13^C_3_]Neu5Ac** | **1,7-lactone/ [^13^C_3_]Neu5Ac** |
| --- | --- | --- | --- |
|  |  |  |  |
| 0.1 | 8.82E+04 ±  8.32E+03 | 2.96E+06 ±  1.30E+05 | 0.030 ± 0.004 |
| 0.25 | 2.60E+05 ±  1.64E+04 | 3.05E+06 ±  5.94E+04 | 0.085 ± 0.007 |
| 0.5 | 5.47E+05 ±  3.40E+04 | 2.86E+06 ±  1.19E+05 | 0.192 ± 0.020 |
| 1 | 9.91E+05 ±  4.86E+04 | 2.88E+06 ±  2.55E+04 | 0.345 ± 0.020 |
| 5 | 6.40E+06 ±  2.86E+05 | 2.89E+06 ±  9.55E+04 | 2.222 ± 0.173 |

**Table S2** Peak area/Concentration curve derived from different concentrations of 1,7-lactone in CH_3_CN/water, 1:1 v/v mixture. The curve is represented by the equation (y=a+bx). Linear regression without using a weighting factor has been applied. C nom is nominal concentration; C calc is the concentration calculated using the derived equation; RE is the relative error expressed in percentage; bias is the mean of the two main errors expressed in percentage.

| **No weight** | C nom | C calc | % RE | % bias |
| --- | --- | --- | --- | --- |
| **b =** 0.450847  **a =** -0.04281  **R^2^ =** 0.997 | 0.1 | 0.168 | 67.71 |  |
|  | 0.1 | 0.155 | 54.77 | **61.236** |
|  | 0.25 | 0.295 | 18.20 |  |
|  | 0.25 | 0.273 | 9.35 | **13.773** |
|  | 0.5 | 0.553 | 10.60 |  |
|  | 0.5 | 0.490 | -1.91 | **4.343** |
|  | 1 | 0.891 | -10.90 |  |
|  | 1 | 0.828 | -17.17 | **-14.033** |
|  | 5 | 5.294 | 5.88 |  |
|  | 5 | 4.752 | -4.96 | **0.459** |

**Table S3** Peak area/Concentration curve derived from different concentrations of 1,7-lactone in CH_3_CN/water, 1:1 v/v mixture. The curve is represented by the equation (y=a+bx). Linear regression with a 1/x^2^ weighting factor has been applied. C nom is nominal concentration; C calc is the concentration calculated using the derived equation; RE is the relative error expressed in percentage; bias is the mean of the two main errors expressed in percentage

| **Weight 1/x^2** | C nom | C calc | % RE | % bias |
| --- | --- | --- | --- | --- |
| **b =** 0.400888  **a =** -0.01105  **R^2^ =** 0.990 | 0.1 | 0.109 | 9.37 |  |
|  | 0.1 | 0.095 | -5.18 | **2.094** |
|  | 0.25 | 0.253 | 1.23 |  |
|  | 0.25 | 0.228 | -8.72 | **-3.743** |
|  | 0.5 | 0.543 | 8.54 |  |
|  | 0.5 | 0.472 | -5.54 | **1.499** |
|  | 1 | 0.923 | -7.72 |  |
|  | 1 | 0.852 | -14.77 | **-11.244** |
|  | 5 | 5.874 | 17.49 |  |
|  | 5 | 5.265 | 5.30 | **11.393** |

**Table S4** Quantification of Neu5Ac, 1,7 lactone and γ-lactone after the purification protocol has been applied starting from three different matrices/media: water, 5% BSA and plasma and using or not the SpeedVac method (40°C x 1.5 h). Neu5Ac or 1,7 lactone or γ-lactone quantification has been expressed as the ratio of the analyte peak area normalized for [^13^C_3_]Neu5Ac and calculated with both the full scan and the PRM acquisition modes. Each value represents the mean of three independent experiments carried out in duplicate.

| **Matrix/**  **Media** | **Drying step** | **Full Scan Experiment** | | | **PRM Experiment** | | |
| --- | --- | --- | --- | --- | --- | --- | --- |
|  |  | **Neu5Ac/** [^13^C_3_]Neu5Ac | **1.7-lactone/**  [^13^C_3_]Neu5Ac | **γ-lactone/** [^13^C_3_]Neu5Ac | **Neu5Ac/** [^13^C_3_]Neu5Ac | **1.7-lactone/** [^13^C_3_]Neu5Ac | **γ-lactone/** [^13^C_3_]Neu5Ac |
| Water | No SpeedVac | - | 0.49 ± 0.10 | 0.12 ± 0.03 | - | 0.81 ± 0.14 | 0.16 ± 0.03 |
|  | SpeedVac | 0.05 ± 0.05 | 0.35 ± 0.05 | 0.13 ± 0.01 | 0.03 ± 0.03 | 0.53 ± 0.09 | 0.14 ± 0.02 |
| 5% BSA | No SpeedVac | 0.07 ± 0.03 | 0.41 ± 0.04 | 0.14 ± 0.01 | - | 0.59 ± 0.09 | 0.17 ± 0.04 |
|  | SpeedVac | 0.09 ± 0.03 | 0.10 ± 0.02 | 0.30 ± 0.10 | 0.06 ± 0.01 | 0.11 ± 0.04 | 0.36 ± 0.14 |
| Plasma | No SpeedVac | 1.34 ± 0.22  (0.35 ± 0.02)^a^ | - | - | 1.29 ± 0.13  (0.32 ± 0.05) ^a^ | - | - |
|  | SpeedVac | 1.73 ± 0.07  (0.34 ± 0.01) ^a^ | - | - | 1.79 ± 0.09  (0.33 ± 0.05)^a^ | - | - |

^a^Ratio referred to the endogenous Neu5Ac levels.


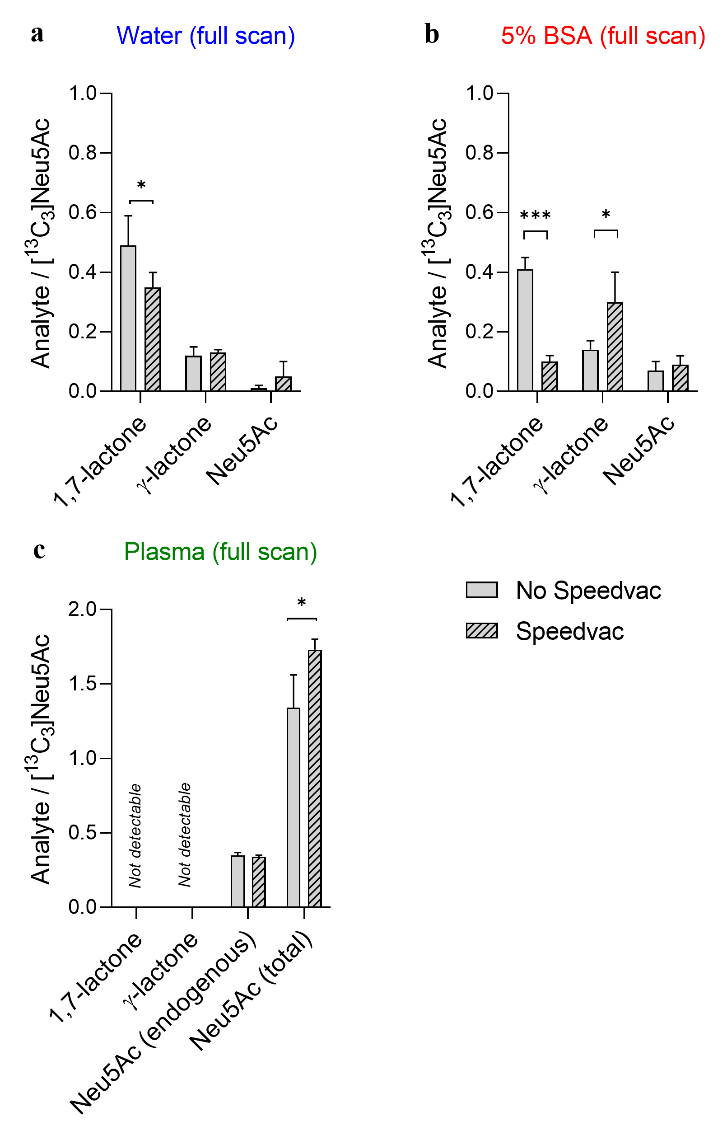


**Fig.S3** Variations of the 1,7-lactone of Neu5Ac, γ-lactone of Neu5Ac and Neu5Ac levels before and after SpeedVac treatment considering the sample processing in different media/matrices: (a) water. (b) 5% BSA. (c) plasma. The levels of analyte have been expressed as the ratio between the analyte and the [^13^C_3_]Neu5Ac peak areas from full scan mode analysis. For plasma also endogenous levels of Neu5Ac have been reported. Each value represents the mean of three independent experiments carried out in duplicate ± standard deviation. A p-value <0.05 has been considered statistically significant; *p-value < 0.05; ***p-value<0.0005
